# Supplementary material for: Use of Publication Dynamics to Distinguish Cancer Genes and Bystander Genes
Source: Genes (Basel). 2022 Jun 21;13(7):1105. doi: 10.3390/genes13071105 (PMC9315931; doi:10.3390/genes13071105)
Supplement: Supplementary file 1 [file genes-13-01105-s001.zip › Appendix SA.pdf]

Appendix SA

The file contains brief description of genes identified in the present study as genes with the highest CA_t values. The majority of these genes are cancer genes that are novel in the sense that they are not included in the most widely used cancer gene lists (***Vogelstein et al. 2013; Sondka et al., 2018***). The assignment of genes to key cellular processes of carcinogenesis is summarized in ***Table 1*** of the main text.

Broad substrate specificity ATP-binding cassette transporter ABCG2, encoded by the ABCG2 gene

ABCG2 is a broad substrate specificity ATP-dependent transporter of the ATP-binding cassette family that actively extrudes a variety of physiological compounds, dietary toxins and xenobiotics from cells. Expression of *ABCG2* can be found in brain, liver, lung cancers and acute myeloid leukemia. It is also expressed at high levels in stem cells and many cell lines (Mehendale-Munj *et al.*, 2021). Over-expression of *ABCG2* in cell lines confers resistance to anticancer drugs including mitoxantrone, daunorubicin, doxorubicin, topotecan and epirubicin. The expression of *ABCG2* has been implicated in multidrug resistance of acute myeloid leukemia and some solid tumors (Han *et al.*, 2004).

Han B, Zhang JT. Multidrug resistance in cancer chemotherapy and xenobiotic protection mediated by the half ATP-binding cassette transporter ABCG2. Curr Med Chem Anticancer Agents. 2004 Jan;4(1):31-42. doi: 10.2174/1568011043482205. PMID: 14754410

Mehendale-Munj S, Sawant S. Breast Cancer Resistance Protein: A Potential Therapeutic Target for Cancer. Curr Drug Targets. 2021;22(4):420-428. doi: 10.2174/1389450121999201125200132. PMID: 33243119

Aldehyde dehydrogenase 1A1, encoded by the ALDH1A1 gene

Aldehyde dehydrogenase 1A1 catalyzes the oxidation of various aldehydes to carboxylic acids, it catalyzes the oxidation of retinaldehyde into retinoic acid.

ALDH1 is a marker of normal and malignant human mammary stem cells and a predictor of poor clinical outcome (Ginestier *et al.,* 2007). Breast cancer cells expressing elevated levels of ALDH1 are displaying the highest tumorigenic potential (Nakshatri *et al.,* 2009). *ALDH1* expression plays an important role in bladder cancer development and prognosis. In patients with invasive bladder cancer, ALDH1 protein expression is significantly associated with an advanced tumor grade, stage, as well as lymph node and distant metastases (Xu *et al*., 2015). In general, high ALDH1 activity and ALDH1A1 overexpression are associated with poor cancer prognosis (Tomita *et al*., 2016).

Ginestier C, Hur MH, Charafe-Jauffret E, Monville F, Dutcher J, Brown M, Jacquemier J, Viens P, Kleer CG, Liu S, Schott A, Hayes D, Birnbaum D, Wicha MS, Dontu G. ALDH1 is a marker of normal and malignant human mammary stem cells and a predictor of poor clinical outcome. Cell Stem Cell. 2007 Nov;1(5):555-67. doi: 10.1016/j.stem.2007.08.014. PMID: 18371393; PMCID: PMC2423808

Nakshatri H, Srour EF, Badve S. Breast cancer stem cells and intrinsic subtypes: controversies rage on. Curr Stem Cell Res Ther. 2009 Jan;4(1):50-60. doi: 10.2174/157488809787169110. PMID: 19149630

Tomita H, Tanaka K, Tanaka T, Hara A. Aldehyde dehydrogenase 1A1 in stem cells and cancer. Oncotarget. 2016 Mar 8;7(10):11018-32. doi: 10.18632/oncotarget.6920. PMID: 26783961; PMCID: PMC4905455

Xu N, Shao MM, Zhang HT, Jin MS, Dong Y, Ou RJ, Wang HM, Shi AP. Aldehyde dehydrogenase 1 (ALDH1) expression is associated with a poor prognosis of bladder cancer. Cancer Epidemiol. 2015 Jun;39(3):375-81. doi: 10.1016/j.canep.2015.03.003. Epub 2015 Apr 3. PMID: 25843691.

Baculoviral IAP repeat-containing protein 5, encoded by the BIRC5 gene

BIRC5 (Survivin) has dual roles in promoting cell proliferation and preventing apoptosis. The protein is essential for chromosome alignment and segregation during mitosis.

BIRC5 protein is prominently expressed in transformed cell lines and most human cancers of lung, colon, pancreas, prostate and breast (Ambrosini *et al*., 1997). BIRC5 is an anti-apoptotic molecule, promoting cell division and tumor progression (Frazzi *et al.,* 2021). Overexpression of *BIRC5* is associated with the initiation and progression of several cancer types (Fäldt Beding *et al.,* 2022).

Ambrosini G, Adida C, Altieri DC. A novel anti-apoptosis gene, survivin, expressed in cancer and lymphoma. Nat Med. 1997 Aug;3(8):917-21. doi: 10.1038/nm0897-917. PMID: 9256286).

Fäldt Beding A, Larsson P, Helou K, Einbeigi Z, Parris TZ. Pan-cancer analysis identifies BIRC5 as a prognostic biomarker. BMC Cancer. 2022 Mar 25;22(1):322. doi: 10.1186/s12885-022-09371-0. PMID: 35331169; PMCID: PMC8953143

Frazzi R. BIRC3 and BIRC5: multi-faceted inhibitors in cancer. Cell Biosci. 2021 Jan 7;11(1):8. doi: 10.1186/s13578-020-00521-0. PMID: 33413657; PMCID: PMC7792207

Polycomb complex protein BMI-1, encoded by the BMI1 gene

BMI1 is a component of a multiprotein complex required to maintain the transcriptionally repressive state of many genes, including Hox genes.

BMI1 plays critical roles in maintaining the self-renewal of hematopoietic, neural, intestinal stem cells, and cancer stem cells for a variety of cancer types. BMI1 represses multiple gene loci through mono-ubiquitination of histone H2A (Lin *et al*., 2015). BMI1 has been shown to play critical roles in prostate self-renewal of cancer repopulating cells and resistance to chemotherapy, resulting in poorer prognosis (Crea *et al*., 2014). Overexpression of *BMI1* is

observed in various cancer types and correlates with advanced stages of disease, aggressive clinicopathological behavior, poor prognosis, resistance to radiation and chemotherapy. Experimental reduction of BMI protein level in tumor cells results in inhibition of cell proliferation, induction of apoptosis and/or senescence, and increases susceptibility to cytotoxic agents and radiation therapy (Janaki Ramaiah and Vaishnave, 2018).

Crea F, Clermont PL, Mai A, Helgason CD. Histone modifications, stem cells and prostate cancer. Curr Pharm Des. 2014;20(11):1687-97. doi: 10.2174/13816128113199990522. PMID: 23888964.

Lin X, Ojo D, Wei F, Wong N, Gu Y, Tang D. A Novel Aspect of Tumorigenesis-BMI1 Functions in Regulating DNA Damage Response. Biomolecules. 2015 Dec 1;5(4):3396-415. doi: 10.3390/biom5043396. PMID: 26633535; PMCID: PMC4693283

Janaki Ramaiah M, Vaishnave S. BMI1 and PTEN are key determinants of breast cancer therapy: A plausible therapeutic target in breast cancer. Gene. 2018 Dec 15;678:302-311. doi: 10.1016/j.gene.2018.08.022. Epub 2018 Aug 8. PMID: 30096458.).

Carbonic anhydrase 9, encoded by the CA9 gene

Carbonic anhydrase 9 catalyzes the reversible hydration of carbon dioxide and participates in pH regulation.

Carbonic anhydrase 9 and 12 are overexpressed in cancer and contribute to tumor physiology. *CA9* is confined to few normal tissues but is ectopically induced in many tumor types due to its strong transcriptional activation by hypoxia, via HIF-1 transcription factor (Pastorekova *et al*., 2006). Carbonic anhydrase 9 is functionally linked to acidosis, implicated in invasiveness, and correlated with therapeutic resistance (Pastorekova *et al*., 2019). Expression of CA9 is linked to poor prognosis in a number of human tumors and may be a marker of aggressive malignant phenotype and a mechanism of progression (Potter *et al*. 2003).

Pastorekova S, Parkkila S, Zavada J. Tumor-associated carbonic anhydrases and their clinical significance. Adv Clin Chem. 2006;42:167-216. PMID: 17131627

Pastorekova S, Gillies RJ. The role of carbonic anhydrase IX in cancer development: links to hypoxia, acidosis, and beyond. Cancer Metastasis Rev. 2019 Jun;38(1-2):65-77. doi: 10.1007/s10555-019-09799-0. PMID: 31076951; PMCID: PMC6647366

Potter CP, Harris AL. Diagnostic, prognostic and therapeutic implications of carbonic anhydrases in cancer. Br J Cancer. 2003 Jul 7;89(1):2-7. doi: 10.1038/sj.bjc.6600936. PMID: 12838292; PMCID: PMC2394207

G2/mitotic-specific cyclin-B1, encoded by the CCNB1 gene

G2/mitotic-specific cyclin-B1 is essential for the control of the cell cycle at the G2/M transition.

The majority of colorectal cancers express high levels of cyclin B1, consistent with a high rate of cell proliferation (Wang *et al.,* 1997). Nuclear cyclin B1 is significantly associated with tumor size, lymph node metastasis and histological grade. Only nuclear cyclin B1 is significantly associated with adverse clinical outcome of the patients, and multivariate analyses of disease-free and overall survival demonstrated nuclear cyclin B1 as the independent marker (Suzuki *et al*., 2007)

Cyclin B1 was identified as a tumor antigen recognized by human T-cells. It is overexpressed at protein and mRNA level in many tumor cell lines including breast, lung, colorectal carcinoma, lymphoma and leukemia. It has been shown that in colorectal tumor cell lines with deleted p53 cyclin B1 is overexpressed, suggesting that p53 plays an important role in cyclin B1 regulation and that tumors with mutated p53 will be good candidates for cyclin B1 based immunotherapy (Yu *et al.,* 2002).

Suzuki T, Urano T, Miki Y, Moriya T, Akahira J, Ishida T, Horie K, Inoue S, Sasano H. Nuclear cyclin B1 in human breast carcinoma as a potent prognostic factor. Cancer Sci. 2007 May;98(5):644-51. doi: 10.1111/j.1349-7006.2007.00444.x. Epub 2007 Mar 14. PMID: 17359284

Wang A, Yoshimi N, Ino N, Tanaka T, Mori H. Overexpression of cyclin B1 in human colorectal cancers. J Cancer Res Clin Oncol. 1997;123(2):124-7. doi: 10.1007/BF01269891. PMID: 9030252

Yu M, Zhan Q, Finn OJ. Immune recognition of cyclin B1 as a tumor antigen is a result of its overexpression in human tumors that is caused by non-functional p53. Mol Immunol. 2002 May;38(12-13):981-7. doi: 10.1016/s0161-5890(02)00026-3. PMID: 12009577).

T-cell antigen CD7, encoded by the CD7 gene

T-cell antigen CD7 is found on thymocytes and mature T cells. It plays an essential role in T-cell interactions and in T-cell/B-cell interaction during early lymphoid development.

The *CD7* antigen loss was frequently observed in aggressive natural killer-cell leukemia (ANKL) patients. In conjunction with the cytogenetic findings, this characteristic immunophenotypic finding can serve as a reliable marker for the timely diagnosis of ANKL therefore immunophenotypic analysis of CD7 expression is included in the diagnosis of NK cell neoplasms (Yoo *et al.*, 2009)

Yoo EH, Kim HJ, Lee ST, Kim WS, Kim SH. Frequent CD7 antigen loss in aggressive natural killer-cell leukemia: a useful diagnostic marker. Korean J Lab Med. 2009 Dec;29(6):491-6. doi: 10.3343/kjlm.2009.29.6.491. PMID: 20046078

Signal transducer CD24, encoded by the CD24 gene

CD24 modulates B-cell activation responses, promotes antigen-dependent proliferation of B-cells, and prevents their terminal differentiation into antibody-forming cells.

High rates of CD24 expression have been found in epithelial ovarian cancer, breast cancer, non-small cell lung cancer, prostate cancer and pancreatic cancer. High rates of CD24 are significantly associated with a more aggressive course of the disease and shorter patient survival times (Kristiansen *et al*., 2004). A high level of CD24 expression is observed in adenocarcinomas, patients whose tumors have a high CD24 expression show a significantly shorter median survival time. The decreased survival of non-small cell lung cancer patients with strongly CD24-positive tumors is related to an enhanced propensity of haematogenous metastasis formation (Kristiansen *et al*., 2003). *CD24* is a potential oncogene reported to be overexpressed in a large variety of human malignancies. Anti-CD24 monoclonal antibodies induce a significant growth inhibition in colorectal and pancreatic cancer cell lines that express the protein. Down-regulation of CD24 retarded tumorigenicity of human cancer cell lines in nude mice (Sagiv *et al.*, 2008).

Kristiansen G, Schlüns K, Yongwei Y, Denkert C, Dietel M, Petersen I. CD24 is an independent prognostic marker of survival in nonsmall cell lung cancer patients. Br J Cancer. 2003 Jan 27;88(2):231-6. doi: 10.1038/sj.bjc.6600702. PMID: 12610508; PMCID: PMC2377041

Kristiansen G, Sammar M, Altevogt P. Tumour biological aspects of CD24, a mucin-like adhesion molecule. J Mol Histol. 2004 Mar;35(3):255-62. doi: 10.1023/b:hijo.0000032357.16261.c5. PMID: 15339045

Sagiv E, Starr A, Rozovski U, Khosravi R, Altevogt P, Wang T, Arber N. Targeting CD24 for treatment of colorectal and pancreatic cancer by monoclonal antibodies or small interfering RNA. Cancer Res. 2008 Apr 15;68(8):2803-12. doi: 10.1158/0008-5472.CAN-07-6463. PMID: 18413748

CD99 antigen, encoded by the CD99 gene

CD99 (MIC2) antigen is involved in T-cell adhesion processes and in spontaneous rosette formation with erythrocytes. It plays a role in leukocyte extravasation through the endothelial basement membrane.

Ewing's sarcoma and peripheral primitive neuroectodermal tumor cells express the *MIC2 (CD99)* gene in very high amounts (Ambros *et al*., 1991). CD99 shows strong expression in lymphoblastic lymphomas and related leukemias (Riopel *et al.,* 1994). Knocking down CD99 expression in human Ewing's sarcoma cell lines reduced their ability to form tumors and bone metastases when xenografted into immunodeficient mice. CD99 inhibits neural differentiation of human Ewing sarcoma cells and thereby contributes to oncogenesis (Rocchi *et al.,* 2010). CD99 is more highly expressed in malignant glioma than in normal brain and the overexpression of CD99 significantly increases invasiveness and decreases survival rate (Seol *et al.,* 2012).

Ambros IM, Ambros PF, Strehl S, Kovar H, Gadner H, Salzer-Kuntschik M. MIC2 is a specific marker for Ewing's sarcoma and peripheral primitive neuroectodermal tumors. Evidence for a common histogenesis of Ewing's sarcoma and peripheral primitive neuroectodermal tumors from MIC2 expression and specific chromosome aberration. Cancer. 1991 Apr 1;67(7):1886-93. doi: 10.1002/1097-0142(19910401)67:7<1886::aid-cncr2820670712>3.0.co;2-u. PMID: 1848471

Riopel M, Dickman PS, Link MP, Perlman EJ. MIC2 analysis in pediatric lymphomas and leukemias. Hum Pathol. 1994 Apr;25(4):396-9. doi: 10.1016/0046-8177(94)90149-x. PMID: 8163272

Rocchi A, Manara MC, Sciandra M, Zambelli D, Nardi F, Nicoletti G, Garofalo C, Meschini S, Astolfi A, Colombo MP, Lessnick SL, Picci P, Scotlandi K. CD99 inhibits neural differentiation of human Ewing sarcoma cells and thereby contributes to oncogenesis. J Clin Invest. 2010 Mar;120(3):668-80. doi: 10.1172/JCI36667. Epub 2010 Feb 8. PMID: 20197622; PMCID: PMC2827943

Seol HJ, Chang JH, Yamamoto J, Romagnuolo R, Suh Y, Weeks A, Agnihotri S, Smith CA, Rutka JT. Overexpression of CD99 Increases the Migration and Invasiveness of Human Malignant Glioma Cells. Genes Cancer. 2012 Sep;3(9-10):535-49. doi: 10.1177/1947601912473603. PMID: 23486730; PMCID: PMC3591096

M-phase inducer phosphatase 3, encoded by the CDC25C gene

CDC25C is a tyrosine protein phosphatase required for progression of the cell cycle. When phosphorylated, it activates G2 cells into prophase. CDC25C dephosphorylates CDK1 and activates its kinase activity.

CDC25C is highly expressed in lung, liver, gastric, bladder, prostate, esophageal and colorectal cancers. Downregulation of CDC25C induces cell cycle arrest in G2/M phase and its abnormal expression is associated with cancer initiation, development, metastasis, occurrence and poor prognosis (Liu *et al*., 2020). CDC25C plays a crucial role in the pathogenesis and/or progression of vulvar and prostate carcinomas (Ozen *et al*., 2005; Wang *et al*., 2010).

Liu K, Zheng M, Lu R, Du J, Zhao Q, Li Z, Li Y, Zhang S. The role of CDC25C in cell cycle regulation and clinical cancer therapy: a systematic review. Cancer Cell Int. 2020 Jun 3;20:213. doi: 10.1186/s12935-020-01304-w. PMID: 32518522; PMCID: PMC7268735

Wang Z, Trope CG, Flørenes VA, Suo Z, Nesland JM, Holm R. Overexpression of CDC25B, CDC25C and phospho-CDC25C (Ser216) in vulvar squamous cell carcinomas are associated with malignant features and aggressive cancer phenotypes. BMC Cancer. 2010 May 25;10:233. doi: 10.1186/1471-2407-10-233. PMID: 20500813; PMCID: PMC2887779

Ozen M, Ittmann M. Increased expression and activity of CDC25C phosphatase and an alternatively spliced variant in prostate cancer. Clin Cancer Res. 2005 Jul 1;11(13):4701-6. doi: 10.1158/1078-0432.CCR-04-2551. PMID: 16000564

Cyclin-dependent kinase 4 inhibitor B, encoded by the CDKN2B gene

CDKs, or cyclin-dependent kinases, are heteromeric serine/threonine kinases that control progression through the cell cycle, transcription, and neuronal function and development.

CDKN2B, a cyclin-dependent kinase inhibitor, is a tumor suppressor gene inhibiting cell cycle progression.

The CpG island inside the *CDKN2B* promoter is hyper-methylated in ~50 – 60% of acute promyelocytic leukemia patients. *CDKN2B* methylation correlates negatively with disease-free-survival (Chim CS, Kwong YL, 2006). Deletions in the 9p21.3 cluster involving *CDKN2A/ARF/CDKN2B* genes arise as one of the oncogenic hallmarks of acute lymphoblastic leukemia. Deletions in this region are the most frequent structural alteration in T-cell acute lymphoblastic leukemia and account for roughly 30% of copy number alterations found in B-cell-precursor acute lymphoblastic leukemia (González-Gil *et al.*, 2021).

Chim CS, Kwong YL. Adverse prognostic impact of CDKN2B hyper-methylation in acute promyelocytic leukemia. Leuk Lymphoma. 2006 May;47(5):815-25. doi: 10.1080/10428190500513827. PMID: 16753865

González-Gil C, Ribera J, Ribera JM, Genescà E. The Yin and Yang-Like Clinical Implications of the CDKN2A/ARF/CDKN2B Gene Cluster in Acute Lymphoblastic Leukemia. Genes (Basel). 2021 Jan 9;12(1):79. doi: 10.3390/genes12010079. PMID: 33435487; PMCID: PMC7827355

Transcription factor E2F1, encoded by the E2F1 gene

Transcription factor E2F1 binds to the promoter region of a number of genes whose products are involved in cell cycle regulation or in DNA replication.

E2F-1 can induce both apoptosis and S-phase transition. It acts as an oncogene or a tumor-suppressor gene depending on the extent to which E2F-1 induces apoptosis as opposed to G1/S transition (Macleod, 1999). Mice lacking *E2F-1* develop a broad spectrum of tumors. Although overexpression of E2F-1 in tissue culture cells can stimulate cell proliferation and be

oncogenic, loss of *E2F-1* in mice results in tumorigenesis, demonstrating that E2F-1 also functions as a tumor suppressor (Yamasaki *et al*., 1996).

Macleod K. pRb and E2f-1 in mouse development and tumorigenesis. Curr Opin Genet Dev. 1999 Feb;9(1):31-9. doi: 10.1016/s0959-437x(99)80005-7. PMID: 10072353

Yamasaki L, Jacks T, Bronson R, Goillot E, Harlow E, Dyson NJ. Tumor induction and tissue atrophy in mice lacking E2F-1. Cell. 1996 May 17;85(4):537-48. doi: 10.1016/s0092-8674(00)81254-4. PMID: 8653789

DNA excision repair protein ERCC-1, encoded by the ERCC1 gene

DNA excision repair protein ERCC-1 is the non-catalytic component of a DNA repair endonuclease responsible for the 5'-incision during DNA repair.

ERCC1 expression levels in human tumor tissue may have a role in clinical resistance to platinum compounds. Patients who were clinically resistant to platinum-based therapy had a 2.6-fold higher expression level of ERCC1 in their tumor tissue than did patients who responded to that therapy (Dabholkar *et al.,* 1992)

Dabholkar M, Bostick-Bruton F, Weber C, Bohr VA, Egwuagu C, Reed E. ERCC1 and ERCC2 expression in malignant tissues from ovarian cancer patients. J Natl Cancer Inst. 1992 Oct 7;84(19):1512-7. doi: 10.1093/jnci/84.19.1512. PMID: 1433335

Glutamate carboxypeptidase 2, encoded by the FOLH1 gene

Glutamate carboxypeptidase 2 (also known as Folate hydrolase 1, FOLH1 and Prostate-specific membrane antigen, PSMA) has folate hydrolase activity. It is used as a diagnostic and prognostic indicator of prostate cancer.

The expression of glutamate carboxypeptidase 2 mRNA is almost entirely prostate specific in human tissues. Normal and malignant prostatic tissues consistently show high PSMA expression (Silver *et al*., 1997) and the invasive ability of prostate cancer may be modulated by folate levels (Yao *et al.,* 2008). The level of expression of PSMA is higher in carcinoma cells from hormone-refractory patients than in the cells of those who showed a good response to hormonal therapy, suggesting that expression of PSM-specific transcripts is closely associated with malignant transformation of the prostate (Kawakami *et al*., 1997). PSMA is abundantly expressed in the endothelium of vessels of glioblastoma multiforme tissues but not in vessels of normal tissues. Overexpression of PSMA has been shown to promote proliferation, invasion and tube formation ability of human umbilical vein endothelial cells, suggesting that it is a critical regulator in angiogenesis and progression of glioblastoma multiforme (Gao *et al*., 2021).

Gao Y, Zheng H, Li L, Feng M, Chen X, Hao B, Lv Z, Zhou X, Cao Y. Prostate-Specific Membrane Antigen (PSMA) Promotes Angiogenesis of Glioblastoma Through Interacting With ITGB4 and Regulating NF-κB Signaling Pathway. Front Cell Dev Biol. 2021 Mar 4;9:598377. doi: 10.3389/fcell.2021.598377. PMID: 33748101; PMCID: PMC7969793

Kawakami M, Nakayama J. Enhanced expression of prostate-specific membrane antigen gene in prostate cancer as revealed by in situ hybridization. Cancer Res. 1997 Jun 15;57(12):2321-4. PMID: 9192800

Silver DA, Pellicer I, Fair WR, Heston WD, Cordon-Cardo C. Prostate-specific membrane antigen expression in normal and malignant human tissues. Clin Cancer Res. 1997 Jan;3(1):81-5. PMID: 9815541

Yao V, Parwani A, Maier C, Heston WD, Bacich DJ. Moderate expression of prostate-specific membrane antigen, a tissue differentiation antigen and folate hydrolase, facilitates prostate carcinogenesis. Cancer Res. 2008 Nov 1;68(21):9070-7. doi: 10.1158/0008-5472.CAN-08-2328. PMID: 18974153; PMCID: PMC2748916

Forkhead box protein M1, encoded by the FOXM1 gene

Forkhead box protein M1 is a transcription factor regulating the expression of cell cycle genes essential for DNA replication and mitosis.

FOXM1 is overexpressed in many types of human cancer. It is intimately involved in tumorigenesis, as it contributes to oncogenic transformation and participates in tumor initiation, growth, and progression (Wierstra, 2013). FOXM1 is detected in human skin and is upregulated in basal cell carcinomas. Activation of Shh signaling *via* Gli1 is an important determinant of FOXM1 expression in mammalian cells and the up-regulation of FOXM1 in basal cell carcinomas may be one of the mechanisms whereby Shh signaling exerts its mitogenic effect on basal keratinocytes, leading to the development of this common human cancer (Teh *et al*., 2002). FOXM1 transcription factor is essential for the development of hepatocellular carcinoma (Kalinichenko *et al*., 2004). Expression of FoxM1 is significantly elevated in primary breast cancer (Wonsey *et al*., 2005). FoxM1 is overexpressed in human glioblastomas, contributes to glioma tumorigenicity and stimulates the proliferation of tumor cells during development of lung cancer (Liu *et al*., 2006; Kim *et al*., 2006).

Kalinichenko VV, Major ML, Wang X, Petrovic V, Kuechle J, Yoder HM, Dennewitz MB, Shin B, Datta A, Raychaudhuri P, Costa RH. Foxm1b transcription factor is essential for development of hepatocellular carcinomas and is negatively regulated by the p19ARF tumor suppressor. Genes Dev. 2004 Apr 1;18(7):830-50. doi: 10.1101/gad.1200704. PMID: 15082532; PMCID: PMC387422

Kim IM, Ackerson T, Ramakrishna S, Tretiakova M, Wang IC, Kalin TV, Major ML, Gusarova GA, Yoder HM, Costa RH, Kalinichenko VV. The Forkhead Box m1 transcription factor stimulates the proliferation of tumor cells during development of lung cancer. Cancer Res. 2006 Feb 15;66(4):2153-61. doi: 10.1158/0008-5472.CAN-05-3003. PMID: 16489016.

Liu M, Dai B, Kang SH, Ban K, Huang FJ, Lang FF, Aldape KD, Xie TX, Pelloski CE, Xie K, Sawaya R, Huang S. FoxM1B is overexpressed in human glioblastomas and critically regulates the tumorigenicity of glioma cells. Cancer Res. 2006 Apr 1;66(7):3593-602. doi: 10.1158/0008-5472.CAN-05-2912. PMID: 16585184

Teh MT, Wong ST, Neill GW, Ghali LR, Philpott MP, Quinn AG. FOXM1 is a downstream target of Gli1 in basal cell carcinomas. Cancer Res. 2002 Aug 15;62(16):4773-80. PMID: 12183437

Wierstra I. FOXM1 (Forkhead box M1) in tumorigenesis: overexpression in human cancer, implication in tumorigenesis, oncogenic functions, tumor-suppressive properties, and target of anticancer therapy. Adv Cancer Res. 2013;119:191-419. doi: 10.1016/B978-0-12-407190-2.00016-2. PMID: 23870513

Wonsey DR, Follettie MT. Loss of the forkhead transcription factor FoxM1 causes centrosome amplification and mitotic catastrophe. Cancer Res. 2005 Jun 15;65(12):5181-9. doi: 10.1158/0008-5472.CAN-04-4059. PMID: 15958562

Induced myeloid leukemia cell differentiation protein Mcl-1, encoded by the MCL1 gene

Myeloid leukemia cell differentiation protein Mcl-1is involved in the regulation of apoptosis versus cell survival and in the maintenance of viability.

Mcl-1 plays a critical role in controlling life and death decisions; isoform 1 inhibits apoptosis, isoform 2 promotes apoptosis (Michels *et al.*, 2005). Mcl-1 has been shown to be upregulated in breast cancer, hepatocellular carcinoma tissues, as well as numerous hematological and solid tumor malignancies (Winder and Campbell, 2022; Sieghart *et al.*, 2006; Warr and Shore, 2008). Mcl-1 differs from other members of the Bcl-2 family in having a very short half-life, therefore inhibition of its expression and/or neutralization of its anti-apoptotic function make Mcl-1-dependent cells more susceptible to apoptosis, providing an opportunity to combat several types of cancers (Akgul, 2009)

Akgul C. Mcl-1 is a potential therapeutic target in multiple types of cancer. Cell Mol Life Sci. 2009 Apr;66(8):1326-36. doi: 10.1007/s00018-008-8637-6. PMID: 19099185

Michels J, Johnson PW, Packham G. Mcl-1. Int J Biochem Cell Biol. 2005 Feb;37(2):267-71. doi: 10.1016/j.biocel.2004.04.007. PMID: 15474972

Sieghart W, Losert D, Strommer S, Cejka D, Schmid K, Rasoul-Rockenschaub S, Bodingbauer M, Crevenna R, Monia BP, Peck-Radosavljevic M, Wacheck V. Mcl-1 overexpression in hepatocellular carcinoma: a potential target for antisense therapy. J Hepatol. 2006 Jan;44(1):151-7. doi: 10.1016/j.jhep.2005.09.010. Epub 2005 Oct 25. PMID: 16289418).

Warr MR, Shore GC. Unique biology of Mcl-1: therapeutic opportunities in cancer. Curr Mol Med. 2008 Mar;8(2):138-47. doi: 10.2174/156652408783769580. PMID: 18336294

Winder ML, Campbell KJ. MCL-1 is a clinically targetable vulnerability in breast cancer. Cell Cycle. 2022 Mar 29:1-17. doi: 10.1080/15384101.2022.2054096. Epub ahead of print. PMID: 35349392

Proliferation marker protein Ki-67, encoded by the MKI67 gene

Proliferation marker protein Ki-67 is required to maintain individual mitotic chromosomes dispersed in the cytoplasm following nuclear envelope disassembly.

Ki-67 expression in proliferating cells spatially organises heterochromatin, thereby controlling gene expression (Sobecki *et al*., 2016). Ki-67 expression is correlated with cell proliferation and is a prognostic marker for various cancers. Genetic disruption of Ki-67 in human epithelial breast and colon cancer cells depletes the cancer stem cell niche. Ki-67 null cells had a proliferative disadvantage compared to wild-type controls in colony formation assays and displayed increased sensitivity to various chemotherapies. Maintenance of Ki-67 expression is associated with metastatic/clonogenic potential (Cidado *et al.,* 2016).

Cidado J, Wong HY, Rosen DM, Cimino-Mathews A, Garay JP, Fessler AG, Rasheed ZA, Hicks J, Cochran RL, Croessmann S, Zabransky DJ, Mohseni M, Beaver JA, Chu D, Cravero K, Christenson ES, Medford A, Mattox A, De Marzo AM, Argani P, Chawla A, Hurley PJ, Lauring J, Park BH. Ki-67 is required for maintenance of cancer stem cells but not cell proliferation. Oncotarget. 2016 Feb 2;7(5):6281-93. doi: 10.18632/oncotarget.7057. PMID: 26823390; PMCID: PMC4868756

Sobecki M, Mrouj K, Camasses A, Parisis N, Nicolas E, Llères D, Gerbe F, Prieto S, Krasinska L, David A, Eguren M, Birling MC, Urbach S, Hem S, Déjardin J, Malumbres M, Jay P, Dulic V, Lafontaine DLj, Feil R, Fisher D. The cell proliferation antigen Ki-67 organises heterochromatin. Elife. 2016 Mar 7;5:e13722. doi: 10.7554/eLife.13722. PMID: 26949251; PMCID: PMC4841783

Melanoma antigen recognized by T-cells 1, encoded by the MLANA (MART1) gene

Melanoma antigen recognized by T-cells 1 is involved in melanosome biogenesis by ensuring the stability of GPR143. Plays a vital role in the expression, stability, trafficking, and processing of melanocyte protein PMEL, which is critical for the formation of stage II melanosomes.

Expression of MART1 is restricted to melanoma and melanocyte cell lines and human retina (Kawakami *et al.*, 1994). Melan-A/MART-1 is a melanocytic diagnostic marker; two antibodies (A103 and M2-7C10) have become available to study Melan-A/MART-1 expression on archival material. Both antibodies are useful in the differential diagnosis of melanocytic tumors, especially metastatic tumors (Busam *et al.*, 1999).

Busam KJ, Jungbluth AA. Melan-A, a new melanocytic differentiation marker. Adv Anat Pathol. 1999 Jan;6(1):12-8. doi: 10.1097/00125480-199901000-00002. PMID: 10197235

Kawakami Y, Eliyahu S, Delgado CH, Robbins PF, Rivoltini L, Topalian SL, Miki T, Rosenberg SA. Cloning of the gene coding for a shared human melanoma antigen recognized by autologous T cells infiltrating into tumor. Proc Natl Acad Sci U S A. 1994 Apr 26;91(9):3515-9. doi: 10.1073/pnas.91.9.3515. PMID: 8170938; PMCID: PMC43610

Nucleoside diphosphate kinase A, encoded by the NME1 (NM23) gene

Nucleoside diphosphate kinase A plays a major role in the synthesis of nucleoside triphosphates other than ATP.

The RNA levels of *NM23* gene are reduced in tumor cells of high metastatic potential (Rosengard *et al*., 1989). Transfection of a constitutive murine *nm23-1* expression construct into highly metastatic K-1735 TK murine melanoma cells resulted in a reduced incidence of primary tumor formation, significant reductions in tumor metastatic potential independent of tumor cell growth (Leone *et al*., 1991a). Somatic allelic deletion of *nm23-H1* was observed in human breast, renal, colorectal, and lung carcinoma DNA samples, as compared to DNA from matched normal tissues. A homozygous deletion of *nm23-H1* was observed in a lymph node metastasis of a colorectal carcinoma, indicating that *nm23-H1* can be recessively inactivated. The data identify *nm23-H1* as a novel, independent locus for allelic deletion in human cancer, a characteristic shared with previously described suppressor genes (Leone *et al*., 1991b). Accumulating evidence shows that *NM23-H1* has a dichotomous role in tumor metastasis as a suppressor and promoter. *NM23-H1* acts as a metastatic suppressor as well as a promoter in different types of cancers (Yu *et al*., 2021).

Leone A, Flatow U, King CR, Sandeen MA, Margulies IM, Liotta LA, Steeg PS. Reduced tumor incidence, metastatic potential, and cytokine responsiveness of nm23-transfected melanoma cells. Cell. 1991 Apr 5;65(1):25-35. doi: 10.1016/0092-8674(91)90404-m. PMID: 2013093).

Leone A, McBride OW, Weston A, Wang MG, Anglard P, Cropp CS, Goepel JR, Lidereau R, Callahan R, Linehan WM, et al. Somatic allelic deletion of nm23 in human cancer. Cancer Res. 1991 May 1;51(9):2490-3. PMID: 2015608).

Rosengard AM, Krutzsch HC, Shearn A, Biggs JR, Barker E, Margulies IM, King CR, Liotta LA, Steeg PS. Reduced Nm23/Awd protein in tumour metastasis and aberrant Drosophila development. Nature. 1989 Nov 9;342(6246):177-80. doi: 10.1038/342177a0. PMID: 2509941

Nodal homolog, encoded by the NODAL gene

Nodal homolog, a growth factor of the TGFβ subfamily is essential for mesoderm formation and axial patterning during embryonic development.

Nodal promotes glioblastoma multiforme (GBM) cancer cells proliferation, invasion and may also support the stemness of GBM initiating cells (GICs). Cancer cells commonly switch from the pyruvate pathway to glycolysis by upregulating HIF1α and HIF2α, leading to higher glucose uptake, low oxygen consumption and low mitochondrial metabolism. This process renders them more resistant to the hypoxic condition common in cancer. In GBM, Nodal triggers this metabolic switch by upregulating HIF1α (Nana *et al*., 2015). Nodal protein expression is correlated with expression levels of glucose transporter (Glut)-1, hexokinase (HK)-II, pyruvate dehydrogenase kinase (PDK)-1, the phosphorylation level of pyruvate dehydrogenase (PDH), glucose uptake, and lactate accumulation in human glioma cells. Knockdown of Nodal expression with specific small hairpin RNA reduced Glut-1, HK-II, and PDK-1 expressions and PDH phosphorylation (Lai *et al.,* 2013). Nodal overexpression promoted the proliferation and invasion of tumor cells and inhibited their apoptosis, resembling the effect of TGF-β addition. Downregulation of Nodal expression via transfection with Nodal-specific siRNA in the presence of TGF-β weakened the promoting effect of the latter on glioma cells growth, and transfecting Nodal siRNA alone in the absence of exogenous TGF-β more profoundly inhibited the growth of glioma cells (Sun *et al*., 2014).

Treatment of glioma cell lines with recombinant Nodal (rNodal) increased secretion of matrix metalloproteinase 2 (MMP-2) and cell invasiveness. The ectopic expression of Nodal in GBM glioma cells that expressed Nodal at low level resulted in increased MMP-2 secretion, enhanced cell invasiveness, raised cell proliferation rates in vitro, increased tumor growth in vivo, and was associated with poor survival in a mice xenograft model. In contrast, the knockdown of Nodal expression in U87MG glioma cells with high Nodal expression level had reduced MMP-2 secretion, less cell invasiveness, lower tumor growth in vivo and longer lifespan in mice with U87MG/shNodal cell xenografts. In addition, Nodal knockdown promoted the reversion of malignant glioma cells toward a differentiated astrocytic phenotype (Lee *et al.,* 2010).

Lai JH, Jan HJ, Liu LW, Lee CC, Wang SG, Hueng DY, Cheng YY, Lee HM, Ma HI. Nodal regulates energy metabolism in glioma cells by inducing expression of hypoxia-inducible factor 1α. Neuro Oncol. 2013 Oct;15(10):1330-41. doi: 10.1093/neuonc/not086. Epub 2013 Aug 1. PMID: 23911596; PMCID: PMC3779039

Lee CC, Jan HJ, Lai JH, Ma HI, Hueng DY, Lee YC, Cheng YY, Liu LW, Wei HW, Lee HM. Nodal promotes growth and invasion in human gliomas. Oncogene. 2010 May 27;29(21):3110-23. doi: 10.1038/onc.2010.55. Epub 2010 Apr 12. PMID: 20383200

Nana AW, Yang PM, Lin HY. Overview of Transforming Growth Factor β Superfamily Involvement in Glioblastoma Initiation and Progression. Asian Pac J Cancer Prev. 2015;16(16):6813-23. doi: 10.7314/apjcp.2015.16.16.6813. PMID: 26514451

Sun J, Liu SZ, Lin Y, Cao XP, Liu JM. TGF-β promotes glioma cell growth via activating Nodal expression through Smad and ERK1/2 pathways. Biochem Biophys Res Commun. 2014 Jan 17;443(3):1066-72. doi: 10.1016/j.bbrc.2013.12.097. Epub 2013 Dec 25. PMID: 24370825

Programmed cell death protein 1, encoded by the PDCD1 gene

The Programmed cell death 1 gene (*PDCD1*) encodes a cell surface membrane protein from the immunoglobulin superfamily, whose expression is typically induced in B cells, natural killer (NK) T cells, CD4+/CD8+ T cells, and activated monocytes (Francisco *et al*., 2010).

PDCD1 binds to programmed cell death ligand 1 (PD-L1), which is highly expressed on the surface of cancer cells. Tumor cells may escape host immune surveillance by overexpression of PD-L1. The aberrantly high expression of PD-L1 in tumor microenvironment can be attributed to the primary activation of multiple oncogenic signaling and the secondary induction by inflammatory factors such as IFN-γ (Ai *et al*., 2020). Inflammation-induced PD-L1 expression in the tumor microenvironment frequently leads to PDCD1-mediated T-cell exhaustion, which inhibits the anti-tumor cytotoxic T cell response (Baumeister *et al.,* 2016). Abnormally high PD-L1 expression on tumor cells and antigen-presenting cells in the tumor microenvironment mediates tumor immune escape, thus PD-L1 might play an important role in tumor immune evasion (Blank *et al*., 2005; Jiang *et al*., 2019).

PDCD1 can be used as a prognostic marker in multiple cancers (Miao *et al*., 2020)., High expression of PDCD1 was closely related to better overall survival (OS) and disease-specific survival (DSS) in breast invasive carcinoma, head and neck squamous cell carcinoma, skin cutaneous melanoma, and uterine corpus endometrial carcinoma; have a better disease-free interval (DFI) and progression-free interval (PFI) in several cancer types. Meanwhile, the high level of PDCD1 gene expression was associated with poorer OS, DSS, and PFI in brain lower grade glioma and uveal melanoma; poorer OS in acute myeloid leukemia and kidney renal papillary cell carcinoma; poorer OS and DSS in glioblastoma multiforme; poorer DSS in kidney renal clear cell carcinoma (Miao *et al*., 2020).

Ai L, Xu A, Xu J. Roles of PD-1/PD-L1 Pathway: Signaling, Cancer, and Beyond. Adv Exp Med Biol. 2020;1248:33-59. doi: 10.1007/978-981-15-3266-5_3. PMID: 32185706).

Baumeister SH, Freeman GJ, Dranoff G, Sharpe AH. Coinhibitory Pathways in Immunotherapy for Cancer. Annu Rev Immunol. 2016 May 20;34:539-73. doi: 10.1146/annurev-immunol-032414-112049. Epub 2016 Feb 25. PMID: 26927206

Blank C, Gajewski TF, Mackensen A. Interaction of PD-L1 on tumor cells with PD-1 on tumor-specific T cells as a mechanism of immune evasion: implications for tumor immunotherapy. Cancer Immunol Immunother. 2005 Apr;54(4):307-14. doi: 10.1007/s00262-004-0593-x. Epub 2004 Dec 15. PMID: 15599732

Francisco LM, Sage PT, Sharpe AH. The PD-1 pathway in tolerance and autoimmunity. Immunol Rev. 2010 Jul;236:219-42. doi: 10.1111/j.1600-065X.2010.00923.x. PMID: 20636820; PMCID: PMC2919275).

Jiang Y, Chen M, Nie H, Yuan Y. PD-1 and PD-L1 in cancer immunotherapy: clinical implications and future considerations. Hum Vaccin Immunother. 2019;15(5):1111-1122. doi: 10.1080/21645515.2019.1571892. Epub 2019 Mar 19. PMID: 30888929; PMCID: PMC6605868

Miao Y, Wang J, Li Q, Quan W, Wang Y, Li C, Wu J, Mi D. Prognostic value and immunological role of PDCD1 gene in pan-cancer. Int Immunopharmacol. 2020 Dec;89(Pt B):107080. doi: 10.1016/j.intimp.2020.107080. Epub 2020 Oct 15. PMID: 33069926

Progesterone receptor, encoded by the PGR gene

Progesterone receptor is involved in the regulation of eukaryotic gene expression and affects cellular proliferation and differentiation in target tissues. Depending on the isoform, progesterone receptor may function as transcriptional activator or repressor.

Deletion of one *PGR* allele in cervical epithelium has been found to promote spontaneous cervical cancer. Furthermore, low PGR expression was associated with poor prognosis in young patients with cervical cancer. These discoveries point to *PGR* as a haploinsufficient tumor suppressor gene in the uterine cervix (Park *et al.*, 2021). Copy number loss of the *PGR* gene and

decreased expression of progesterone receptor may account for worse clinical outcomes in some individuals with estrogen receptor α (ERα)-positive breast cancer (Thomas *et al*., 2015).

In the presence of agonist ligands, PGR associates with ERα to direct ERα chromatin binding events within breast cancer cells, resulting in a unique gene expression program that is associated with good clinical outcome. Progesterone inhibited oestrogen-mediated growth of ERα(+) cell line xenografts and primary ERα(+) breast tumor explants, and had increased anti-proliferative effects when coupled with an ERα antagonist. Copy number loss of *PGR*, is a common feature in ERα(+) breast cancers, explaining lower PGR levels in a subset of cases (Mohammed *et al*., 2015). Estrogen receptor α (ERα) and progesterone receptor are crucial prognostic and predictive biomarkers that are usually co-expressed in breast cancer. The loss of PGR expression in ERα(+) breast cancer may signify resistance to endocrine therapy and poorer outcomes (Kunc *et al.,* 2021).

Kunc M, Popęda M, Biernat W, Senkus E. Lost but Not Least-Novel Insights into Progesterone Receptor Loss in Estrogen Receptor-Positive Breast Cancer. Cancers (Basel). 2021 Sep 23;13(19):4755. doi: 10.3390/cancers13194755. PMID: 34638241; PMCID: PMC8507533).

Mohammed H, Russell IA, Stark R, Rueda OM, Hickey TE, Tarulli GA, Serandour AA, Birrell SN, Bruna A, Saadi A, Menon S, Hadfield J, Pugh M, Raj GV, Brown GD, D'Santos C, Robinson JL, Silva G, Launchbury R, Perou CM, Stingl J, Caldas C, Tilley WD, Carroll JS. Progesterone receptor modulates ERα action in breast cancer. Nature. 2015 Jul 16;523(7560):313-7. doi: 10.1038/nature14583. Epub 2015 Jul 8. Erratum in: Nature. 2015 Oct 1;526(7571):144. Serandour, Aurelien A A[Corrected to Serandour, Aurelien A]. PMID: 26153859; PMCID: PMC4650274).

Park Y, Baik S, Ho C, Lin CY, Chung SH. Progesterone Receptor Is a Haploinsufficient Tumor-Suppressor Gene in Cervical Cancer. Mol Cancer Res. 2021 Jan;19(1):42-47. doi: 10.1158/1541-7786.MCR-20-0704. Epub 2020 Nov 2. PMID: 33139507; PMCID: PMC7785602

Thomas C, Gustafsson JÅ. Progesterone receptor-estrogen receptor crosstalk: a novel insight. Trends Endocrinol Metab. 2015 Sep;26(9):453-4. doi: 10.1016/j.tem.2015.08.002. Epub 2015 Aug 12. PMID: 26277479

Ras association domain-containing protein 1, encoded by the RASSF1 gene

Ras association domain-containing protein 1 is required for death receptor-dependent apoptosis.

The *RASSF1* gene is a candidate tumor suppressor gene that was isolated from the 120-kb region of the minimal homozygous deletion region at 3p21.3 in lung and breast cancers. The *RASSF1* locus encodes several major transcripts by alternative promoter selection and alternative mRNA splicing. Aberrant methylation of the *RASSF1A* promoter region is one of the most frequent epigenetic inactivation events detected in human cancer and leads to silencing of *RASSF1A*. Hypermethylation of *RASSF1A* was commonly observed in primary tumors including lung, breast, pancreas, kidney, liver, cervix, nasopharyngeal, prostate, thyroid and other cancers. Inactivation of *RASSF1A* was associated with an advanced tumor stage (e.g. bladder, brain, prostate, gastric tumors) and poor prognosis (e.g. lung, sarcoma and breast cancer). Detection of aberrant *RASSF1A* methylation may serve as a diagnostic and prognostic marker. The functional analyses of RASSF1A reveal an involvement in apoptotic signaling, microtubule stabilization and mitotic progression. The tumor suppressor RASSF1A may act as a negative Ras effector inhibiting cell growth and inducing cell death. Thus, *RASSF1A* may represent an epigenetically inactivated *bona fide* tumor suppressor in human carcinogenesis (Dammann *et al*., 2005).

It has been shown that re-expression of RASSF1A in lung carcinoma cells reduced colony formation, suppressed anchorage-independent growth and inhibited tumor formation in nude mice. These characteristics also indicate a potential role for RASSF1A as a lung tumor suppressor gene (Dammann *et al*., 2000).

Dammann R, Li C, Yoon JH, Chin PL, Bates S, Pfeifer GP. Epigenetic inactivation of a RAS association domain family protein from the lung tumour suppressor locus 3p21.3. Nat Genet. 2000 Jul;25(3):315-9. doi: 10.1038/77083. PMID: 10888881

Dammann R, Schagdarsurengin U, Seidel C, Strunnikova M, Rastetter M, Baier K, Pfeifer GP. The tumor suppressor RASSF1A in human carcinogenesis: an update. Histol Histopathol. 2005 Apr;20(2):645-63. doi: 10.14670/HH-20.645. PMID: 15736067

S-phase kinase-associated protein 2, encoded by the SKP2 gene

S-phase kinase-associated protein 2 is a substrate recognition component of a SCF (SKP1-CUL1-F-box protein) E3 ubiquitin-protein ligase complex that mediates the ubiquitination and subsequent proteasomal degradation of target proteins involved in cell cycle progression, signal transduction and transcription. The protein promotes ubiquitination and destruction of CDH1 (Cadherin-1) in a CK1-dependent manner, thereby regulating cell migration.

Skp2 can ubiquitylate various proteins, including p27Kip1, p57Kip2, p130, Tob1, and c-Myc. Skp2 is mainly involved in degradation of tumor suppressor proteins such as Cdk inhibitors and p130. Skp2, the ubiquitin ligase subunit that targets p27(Kip1) for degradation, is commonly overexpressed in human cancers. p27(Kip1) is a negative regulator of the cell cycle that plays an important role in tumor suppression, therefore loss of p27(Kip1) secondary to enhanced ubiquitin-mediated degradation results in uncontrolled proliferation and promotes tumor progression. Overexpression of Skp2 mRNA and protein levels was observed in many aggressive cancers and was commonly associated with down-regulation of p27(Kip1) levels and loss of tumor differentiation. Skp2 is suggested to be an oncogene since its overexpression has been

observed in many human cancers. Skp2 is an independent prognostic marker for disease-free and overall survival (Hershko, 2008; Wang *et al.*, 2012; Kitagawa *et al.*, 2009).

Skp2 functions as a pro-survival gene in tumor progression. Skp2 is involved in cell proliferation, apoptosis, migration, invasion, angiogenesis, and metastasis of many malignant human tumors and is over-expressed in many types of human cancers, including breast cancer, non-small cell lung cancer, prostate cancer, pancreatic cancer, multiple myeloma, gastric cancer, melanoma, lymphoma, nasopharyngeal carcinoma, and osteosarcoma. Over-expression of Skp2 is often associated with higher grades and a greater malignancy of tumors as well as a poor prognosis of cancers, such as breast cancer, nasopharyngeal carcinoma, rectal cancer treated with chemoradiotherapy, oral squamous cell carcinomas, and non-small-cell lung cancer, which suggests that Skp2 engages in tumorigenic activity and is a promising target for cancer therapy. Skp2 is also involved in cancer-associated drug resistance (Wu *et al.,* 2021).

Hershko DD. Oncogenic properties and prognostic implications of the ubiquitin ligase Skp2 in cancer. Cancer. 2008 Apr 1;112(7):1415-24. doi: 10.1002/cncr.23317. PMID: 18260093;

Kitagawa K, Kotake Y, Kitagawa M. Ubiquitin-mediated control of oncogene and tumor suppressor gene products. Cancer Sci. 2009 Aug;100(8):1374-81. doi: 10.1111/j.1349-7006.2009.01196.x. Epub 2009 May 19. PMID: 19459846).

Wang G, Chan CH, Gao Y, Lin HK. Novel roles of Skp2 E3 ligase in cellular senescence, cancer progression, and metastasis. Chin J Cancer. 2012 Apr;31(4):169-77. doi: 10.5732/cjc.011.10319. Epub 2011 Dec 23. PMID: 22200179; PMCID: PMC3777478;

Wu T, Gu X, Cui H. Emerging Roles of SKP2 in Cancer Drug Resistance. Cells. 2021 May 10;10(5):1147. doi: 10.3390/cells10051147. PMID: 34068643; PMCID: PMC8150781

Zinc finger protein SNAI1, encoded by the SNAI1 gene

Zinc finger protein SNAI1 (Protein snail homolog 1) is involved in induction of epithelial to mesenchymal transition, formation and maintenance of embryonic mesoderm, growth arrest, survival and cell migration.

Several transcription factors, including the Snail/Slug family function as molecular switches for the epithelial to mesenchymal transition (EMT) program. Snail is a zinc-finger transcriptional repressor controlling EMT during embryogenesis and tumor progression. Snail expression correlates with the tumor grade, nodal metastasis of many types of tumor and predicts a poor outcome in patients with metastatic cancer. Snail causes a metabolic reprogramming, bestows tumor cells with cancer stem cell-like traits, and additionally, promotes drug resistance, tumor recurrence and metastasis (Wang *et al*., 2013; Blanco *et al*., 2002).

Snail1, a key inducer of epithelial-mesenchymal transition (EMT), plays a critical role in tumor metastasis. Snail1 is involved in tumor immunosuppression by inducing chemokines and immunosuppressive cells into the tumor microenvironment. In addition, some immune checkpoints, such as programmed death ligand 1 (PD-L1), potentiate Snail1 expression (Tang *et al.,* 2021).

The induction of EMT by TGFβ has been recently linked to the acquisition of tumor initiating stem-like cells characteristics in breast cancer TGFβ induced EMT in epithelial cells through the up-regulation of Snail1 in Smad-dependent signaling. In vivo, the down-regulation of Snail1 reduced tumor growth but was not sufficient to eliminate tumor initiation (Dang *et al*., 2011).

Blanco MJ, Moreno-Bueno G, Sarrio D, Locascio A, Cano A, Palacios J, Nieto MA. Correlation of Snail expression with histological grade and lymph node status in breast carcinomas. Oncogene. 2002 May 9;21(20):3241-6. doi: 10.1038/sj.onc.1205416. PMID: 12082640 .

Dang H, Ding W, Emerson D, Rountree CB. Snail1 induces epithelial-to-mesenchymal transition and tumor initiating stem cell characteristics. BMC Cancer. 2011 Sep 19;11:396. doi: 10.1186/1471-2407-11-396. PMID: 21929801; PMCID: PMC3189192

Tang X, Sui X, Weng L, Liu Y. SNAIL1: Linking Tumor Metastasis to Immune Evasion. Front Immunol. 2021 Nov 30;12:724200. doi: 10.3389/fimmu.2021.724200. PMID: 34917071; PMCID: PMC8669501

Wang Y, Shi J, Chai K, Ying X, Zhou BP. The Role of Snail in EMT and Tumorigenesis. Curr Cancer Drug Targets. 2013 Nov;13(9):963-972. doi: 10.2174/15680096113136660102. PMID: 24168186; PMCID: PMC4004763

Tumor necrosis factor ligand superfamily member 10, encoded by the TNFSF10 gene

Death ligands such as tumor necrosis factor-related apoptosis-inducing ligand (TRAIL/TNFSF10) initiate apoptosis through activation of the extrinsic apoptotic pathway and also exert non-apoptotic biological functions such as regulation of inflammation and cancer metastasis.

TRAIL gene-targeted mice were more susceptible to experimental and spontaneous tumor metastasis and also more sensitive to the chemical carcinogen methylcholanthrene, supporting TRAIL as an important natural effector molecule used in the host defense against transformed cells and cancer cell metastasis. Despite suppressive function of TRAIL/death receptor in the regulation of cancer cell invasion and metastasis, TRAIL has also been reported to strongly increase the distant metastatic spread of pancreatic tumors in vivo. Bioinformatic analyses showed that TRAIL expression leads to differential outcomes of disease-free survival in adenocarcinoma and squamous cell carcinoma (Oh *et al*., 2021).

TRAIL signaling may lead to either anti- or protumorigenic effects. The tumor microenvironment is a source for multiple signals that are able to modulate TRAIL signaling in tumor and stromal cells. Immune effector cells employ the TRAIL/TRAIL-R system whereby

cell surface expressed TRAIL can activate apoptosis via TRAIL receptors on tumor cells, which is part of tumor immune surveillance. The tumor- tumor microenvironment interactions are complex and often bidirectional leading to tumor-enhancing or tumor-reducing effects in a tumor model- and tumor type-dependent fashion. Multiple signals originating from different components of the tumor microenvironment simultaneously affect TRAIL receptor signaling (de Looff *et al.*, 2019).

de Looff M, de Jong S, Kruyt FAE. Multiple Interactions Between Cancer Cells and the Tumor Microenvironment Modulate TRAIL Signaling: Implications for TRAIL Receptor Targeted Therapy. Front Immunol. 2019 Jul 3;10:1530. doi: 10.3389/fimmu.2019.01530. PMID: 31333662; PMCID: PMC6617985

Oh YT, Sun SY. Regulation of Cancer Metastasis by TRAIL/Death Receptor Signaling. Biomolecules. 2021 Mar 26;11(4):499. doi: 10.3390/biom11040499. PMID: 33810241; PMCID: PMC8065657

Vascular endothelial growth factor C, encoded by the VEGFC gene

Vascular endothelial growth factor C is active in angiogenesis and endothelial cell growth, stimulating their proliferation and migration and also has effects on the permeability of blood vessels. May function in angiogenesis of the venous and lymphatic vascular systems during embryogenesis, and also in the maintenance of differentiated lymphatic endothelium in adults.

Increased expression of the protein in primary tumors correlates with increased dissemination of tumor cells to regional lymph nodes in a variety of human carcinomas. The tumor-associated lymphatic VEGFC is a key component of metastatic spread (Pepper, 2001).

The sentinel lymph node (SLN) is the first lymph node in the draining nodal basin to receive cancer cells. Patients with a negative SLN biopsy show a significantly lower incidence of distant metastasis, suggesting that the SLN may be the major gateway for cancer metastasis in these cancer types (Leong *et al*., 2022). Breast cancer usually initially metastases to the SLNs. Recent studies have demonstrated that tumor cells induce SLN lymphangiogenesis before metastasis in several malignancies. In addition, tumor-derived VEGFC or VEGFD may induce lymphangiogenesis and promote lymph node metastasis. It has been shown that primary breast tumors induce SLN lymphangiogenesis before metastasis occurs and that tumor-derived VEGF-C, but not VEGF-D, plays an important role in SLN lymphangiogenesis in breast cancer (Zhao *et al*., 2012a). Another study showed that peritumoral lymphangiogenesis induced by VEGF-C and VEGF-D promotes lymph node metastasis in breast cancer patients (Zhao *et al*., 2012b).

Vascular endothelial growth factor receptor 3 (VEGFR3) has been known for its involvement in tumor-associated lymphangiogenesis and lymphatic metastasis. The VEGFR3 signaling is stimulated by its main cognate ligand, vascular endothelial growth factor C (VEGF-C), which in turn promotes tumor progression. Activation of VEGF-C/VEGFR3 signaling in lymphatic endothelial cells (LECs) was shown to enhance the proliferation of LECs and the formation of lymphatic vessels, leading to increased lymphatic metastasis of tumor cells (Hsu *et al*., 2019).

Hsu MC, Pan MR, Hung WC. Two Birds, One Stone: Double Hits on Tumor Growth and Lymphangiogenesis by Targeting Vascular Endothelial Growth Factor Receptor 3. Cells. 2019 Mar 21;8(3):270. doi: 10.3390/cells8030270. PMID: 30901976; PMCID: PMC6468620).

Leong SP, Naxerova K, Keller L, Pantel K, Witte M. Molecular mechanisms of cancer metastasis via the lymphatic versus the blood vessels. Clin Exp Metastasis. 2022 Feb;39(1):159-179. doi: 10.1007/s10585-021-10120-z. Epub 2021 Nov 12. PMID: 34767139; PMCID: PMC8967809

Pepper MS. Lymphangiogenesis and tumor metastasis: myth or reality? Clin Cancer Res. 2001 Mar;7(3):462-8. PMID: 11297234

Zhao YC, Ni XJ, Wang MH, Zha XM, Zhao Y, Wang S. Tumor-derived VEGF-C, but not VEGF-D, promotes sentinel lymph node lymphangiogenesis prior to metastasis in breast cancer patients. Med Oncol. 2012 Dec;29(4):2594-600. doi: 10.1007/s12032-012-0205-0. Epub 2012 May 6. PMID: 22562155

Zhao YC, Ni XJ, Li Y, Dai M, Yuan ZX, Zhu YY, Luo CY. Peritumoral lymphangiogenesis induced by vascular endothelial growth factor C and D promotes lymph node metastasis in breast cancer patients. World J Surg Oncol. 2012 Aug 20;10:165. doi: 10.1186/1477-7819-10-165. PMID: 22906075; PMCID: PMC3499230

E3 ubiquitin-protein ligase XIAP, encoded by the XIAP gene

E3 ubiquitin-protein ligase XIAP is a multi-functional protein which regulates not only caspases and apoptosis, but also modulates inflammatory signaling and immunity, copper homeostasis, mitogenic kinase signaling, cell proliferation, as well as cell invasion and metastasis. Acts as a direct caspase inhibitor. Acts as an E3 ubiquitin-protein ligase.

X-linked inhibitor of apoptosis protein (XIAP), also known as IAP3, BIRC4, and hILP, belongs to IAP family. Like other human IAPs, XIAP inhibits cell death mainly through blocking apoptosis. XIAP binds to the apoptosis initiator caspase-9 and effector caspase-3/7, marking them for proteosome degradation by ubiquitination. Dysregulation of XIAP has been shown to impact the progression of multiple cancers. XIAP enables cells to undergo malignant transformation, thus preventing cell death and initiating carcinogenesis. XIAP has been found to be involved in many other cell death pathways, including autophagy, necroptosis and copper homeostasis. XIAP should not be regarded only as apoptosis inhibitor, but rather as a master cell death resistance regulator (Tu *et al*., 2020)

Down-regulation of XIAP resulted in caspase-3 activation, caspase-mediated MDM2 processing, and p53 accumulation and induces apoptosis in chemoresistant human ovarian cancer cells (Sasaki *et al*., 2000). It has been shown that the protein XIAP is overexpressed in renal cell carcinoma (RCC), and that high XIAP expression in RCC predicted a worse prognosis. In addition, XIAP antisense oligonucleotide sensitized RCC to Fas/TRAIL-induced apoptosis. These results suggest that XIAP expression in RCC may be used as a prognostic parameter, and that downregulation or inhibition of XIAP expression in RCC may reverse immune resistance (Mizutani *et al*., 2007).

Mizutani Y, Nakanishi H, Li YN, Matsubara H, Yamamoto K, Sato N, Shiraishi T, Nakamura T, Mikami K, Okihara K, Takaha N, Ukimura O, Kawauchi A, Nonomura N, Bonavida B, Miki T. Overexpression of XIAP expression in renal cell carcinoma predicts a worse prognosis. Int J Oncol. 2007 Apr;30(4):919-25. PMID: 17332931).

Sasaki H, Sheng Y, Kotsuji F, Tsang BK. Down-regulation of X-linked inhibitor of apoptosis protein induces apoptosis in chemoresistant human ovarian cancer cells. Cancer Res. 2000 Oct 15;60(20):5659-66. PMID: 11059757).

Tu H, Costa M. XIAP's Profile in Human Cancer. Biomolecules. 2020 Oct 29;10(11):1493. doi: 10.3390/biom10111493. PMID: 33138314; PMCID: PMC7692959

DNA repair protein XRCC1, encoded by the XRCC1 gene

DNA repair protein XRCC1 is involved in DNA single-strand break repair by mediating the assembly of DNA break repair protein complexes.

XRCC1 is a molecular scaffold protein that interacts with multiple enzymatic components of DNA single-strand break repair that collectively are capable of accelerating the

repair of a broad range of DNA single-strand breaks. Hereditary mutations in *XRCC1* have not yet been associated with elevated risk of cancer. In contrast, epidemiological studies have correlated a number of common polymorphisms in *XRCC1* with mildly altered cancer risk, of which R194W, R280H, and R399Q are the most common. Despite being the focus of intense and extensive epidemiological analyses, however, the data linking the polymorphic alleles with altered cancer risk remain highly equivocal (Caldecott *et al*, 2019).

It has been demonstrated that the T allele and CT genotype of *XRCC1* rs1799782 (R194W) polymorphism are associated with an increased risk to develop oral cancer (Mozaffari *et al*., 2021). Studies evaluating the association between *XRCC1* polymorphisms and non-Hodgkin lymphoma (NHL) risk are controversial. The R194W polymorphism was associated with increased NHL risk within the Asian population, and increased diffuse large B cell lymphoma (DLBCL) risk within the overall population under dominant model. The R399Q polymorphism, however, was associated with decreased risk for NHL and DLBCL under heterozygous and dominant models of inheritance (Li *et al*., 2015).

It has been shown that expression of R280H, the most predominant variants of XRCC1 induces cellular transformation in human MCF10A cells. Cells expressing R280H showed significantly increased levels of chromosomal aberrations and accumulate double strand breaks in the G1 cell cycle phase, confirming a possible link between R280H and genomic instability and suggest that individuals carrying this mutation may be at increased risk of cancer development (Sizova *et al*., 2015).

According to the meta-analysis of Liu and Jiang (2017) glioma susceptibility is associated with rs1799782 (R194W) and rs25487 (Q399R) of X-ray repair complementing defective repair in Chinese hamster cells 1 (XRCC1) in Caucasian population.

Caldecott KW. XRCC1 protein; Form and function. DNA Repair (Amst). 2019 Sep;81:102664. doi: 10.1016/j.dnarep.2019.102664. Epub 2019 Jul 8. PMID: 31324530

Li Y, Bai O, Cui J, Li W. Genetic polymorphisms in the DNA repair gene, XRCC1 associate with non-Hodgkin lymphoma susceptibility: A systematic review and meta-analysis. Eur J Med Genet. 2016 Feb;59(2):91-103. doi: 10.1016/j.ejmg.2015.12.011. Epub 2015 Dec 23. PMID: 26723520

Liu K, Jiang Y. Polymorphisms in DNA Repair Gene and Susceptibility to Glioma: A Systematic Review and Meta-Analysis Based on 33 Studies with 15 SNPs in 9 Genes. Cell Mol Neurobiol. 2017 Mar;37(2):263-274. doi: 10.1007/s10571-016-0367-y. Epub 2016 Apr 7. PMID: 27055523

Mozaffari HR, Rostamnia M, Sharifi R, Safaei M, Zavattaro E, Tadakamadla SK, Imani MM, Sadeghi M, Golshah A, Moradpoor H, Rezaei F, Omidpanah N, Hatami M. A PRISMA-compliant meta-analysis on association between X-ray repair cross complementing (XRCC1, XRCC2, and XRCC3) polymorphisms and oral cancer susceptibility. Gene. 2021 May 20;781:145524. doi: 10.1016/j.gene.2021.145524. Epub 2021 Feb 23. PMID: 33631241

Sizova DV, Keh A, Taylor BF, Sweasy JB. The R280H X-ray cross-complementing 1 germline variant induces genomic instability and cellular transformation. DNA Repair (Amst). 2015 Jul;31:73-9. doi: 10.1016/j.dnarep.2015.05.005. Epub 2015 May 14. PMID: 26011397; PMCID: PMC4458331
